# Paradoxical Psoriasis in Patients Receiving Therapy with Tumor Necrosis Factor Inhibitors: Potential Pathogenic Mechanisms and the Role of Genetic Factors

**DOI:** 10.3390/ijms25137018

**Published:** 2024-06-27

**Authors:** Damiana Costin, Alexandra Maria Burlui, Anca Cardoneanu, Luana Andreea Macovei, Ciprian Rezus, Ioana Bratoiu, Patricia Richter, Ioana Ruxandra Mihai, Andreea Gherasim, Ciprian Danielescu, Elena Rezus

**Affiliations:** 1 Department of Medical Sciences II, Faculty of Medicine, “Grigore T. Popa” University of Medicine and Pharmacy, 700115 Iasi, Romania; damiana.costin@umfiasi.ro (D.C.); anca.cardoneanu@umfiasi.ro (A.C.); luana.macovei@umfiasi.ro (L.A.M.); ioana.bratoiu@umfiasi.ro (I.B.); patricia.richter@umfiasi.ro (P.R.); ioana-ruxandra_mihai@umfiasi.ro (I.R.M.); andreea.gherasim@umfiasi.ro (A.G.); elena.rezus@umfiasi.ro (E.R.); 2 Clinical Rehabilitation Hospital, 700661 Iasi, Romania; 3 Department of Internal Medicine, Faculty of Medicine, “Grigore T. Popa” University of Medicine and Pharmacy, 700115 Iasi, Romania; ciprian.rezus@umfiasi.ro; 4 “Sfantul Spiridon” Emergency Hospital, 700111 Iasi, Romania; 5 Department of Surgery II, Faculty of Medicine, “Grigore T. Popa” University of Medicine and Pharmacy, 700115 Iasi, Romania; 6 “Profesor Dr. Nicolae Oblu” Clinical Emergency Hospital, 700309 Iasi, Romania

**Keywords:** paradoxical psoriasis, tumor necrosis factor-alpha inhibitors, genetic polymorphisms, paradoxical adverse effects, genetic predisposition, gene

## Abstract

TNF inhibitors (TNFi) have revolutionized the therapeutic management of various chronic immune-mediated inflammatory diseases. Despite their known benefits, these therapies are related to paradoxical adverse effects (PAEs), including paradoxical psoriasis (PP). Although the underlying mechanism remains somewhat unclear, some theories suggest that genetic factors, particularly certain single-nucleotide polymorphisms (SNPs), may play an important role. The present review aimed to research and analyze recent findings regarding the pathomechanisms involved in the appearance of PP and the association between various genetic factors and PP in individuals treated with TNFi. We performed a literature search and found that certain genes *(IL23R*, *TNF*, *FBXL19*, *CTLA4*, *SLC12A8*, *TAP1*) are strongly associated with the occurrence of PP in pediatric and adult patients during therapy with TNFi. The identification of the specific SNPs involved in the appearance of PP and other PAEs in patients treated with TNFi for various diseases and in different populations may later favor the recognition of those patients at a high risk of developing such adverse effects and could guide personalized therapeutic strategies in future years.

## 1. Introduction

Biological agents, especially tumor necrosis factor inhibitors (TNFi), have revolutionized the therapeutic management of various chronic immune-mediated inflammatory diseases such as rheumatoid arthritis (RA), ankylosing spondylitis (AS), psoriatic arthritis (PA), juvenile idiopathic arthritis (JIA), inflammatory bowel disease (IBD), and psoriasis [1,2,3,4]. They have shown significant results in reducing symptoms, disease activity, and improving the patients’ quality of life [2]. The effectiveness, tolerability, and safety of these treatments are widely acknowledged [1].

It is important to identify new therapeutic targets that restore the immune dysregulation by correcting the imbalance between pro- and anti-inflammatory cytokines in immune-inflammatory diseases. TNFα is an important cytokine involved in the pathogenesis of several chronic immune-mediated inflammatory conditions, and therefore, blocking this signaling pathway was proven effective in the management of such diseases such as RA, PA, AS, IBD, JIA, and psoriasis [5,6,7,8].

Despite the numerous benefits of biological agents, they are related to various adverse reactions, including “paradoxical adverse effects” (PAEs). In 2018, Mylonas et al. estimated that over two million people have received treatment with TNFi, and the number is expected to increase [9], yet PAEs continue to be a cause of concern for clinicians [10].

PAEs are defined as a new development or as the worsening of a pre-existing pathological condition that normally responds to biological agents [1,11]. Several PAEs have been reported in association with TNFi [12] and less often with other biological agents such as tocilizumab, rituximab, ustekinumab, secukinumab, and ixekizumab [13,14,15].

The PAEs associated with TNFi agents are various and include dermatological diseases (psoriasiform skin reaction, alopecia areata, hidradenitis suppurativa (HS), acneiform reactions, and pyoderma gangrenosum), gastroenterological conditions (inflammatory bowel disease), and ophthalmological diseases (uveitis) [1,11,16].

The pathogenesis of these reactions is poorly understood [17], with several theories suggesting that these PAEs are caused by an interaction of various factors, including genetic predisposition, environmental factors, disparity of cytokines levels, and pre-existing autoimmunity. Some theories associate these adverse effects with antibody production against biological agents, considering a hypersensitivity reaction [14]. Ibis et al. suggest that this pathological condition involves some immunological pathways, such as a disparity of cytokines and receptors and an overproduction of interferon-alpha (INFα) [18,19].

Among all the PAEs, paradoxical psoriasis (PP) is the most common cutaneous adverse effect observed in patients treated with biological agents, particularly in those receiving TNFi therapy [3]. According to the BIOGEAS registry (international registry of autoimmune diseases induced by biologics), which contains about 12,731 patients, psoriasis was the most commonly induced autoimmune disease (6375 cases), with 99% being caused by TNFi [20].

PP is an important side effect that represents a serious problem for physicians, sometimes leading to cessation or switching to other biological agents [9].

Recent studies have shown that genetic predisposition may be associated with the development of PP during TNFi therapy [21]. According to the literature, single nucleotide polymorphisms (SNPs) can be associated with the response to biological treatment and also with the development of PP [22,23].

The present review aimed to research and analyze recent findings regarding the association between various SNPs and PP in individuals treated with TNFi. Moreover, we aimed to highlight the implications of these genetic findings depending on the therapeutic agent used (various TNFi).

The search of the published data was performed on the EMBASE, EBSCO, and PubMed Databases, combining the following terms: “paradoxical psoriasis” or “TNF alpha-induced psoriasis”, “psoriasis under TNF inhibitors” or “paradoxical psoriasiform reactions” with “genetic polymorphisms” or “genetic risk” or “SNP” or “biomarkers” or “gene polymorphisms predictors” or “gene”, and “risk factors”. All search terms were repeated with “etanercept”, “infliximab”, “adalimumab”, or “certolizumab” as an alternative to “anti-TNF”. Complete articles were used. No limitations were imposed on language, race, ethnicity, or geographic region.

## 2. Paradoxical Psoriasis Induced by TNFi

### 2.1. Definition and General Considerations

PP is defined as a new onset or exacerbation of pre-existing psoriatic lesions, which may occur in rheumatologic and non-rheumatologic patients treated with biological agents [24]. PP, recognized as a prototypical cutaneous manifestation of PAEs, is a rare immune-mediated, non-infectious, and non-neoplastic inflammatory condition that occurs following TNFi therapy. It is commonly referred to as a class-effect of TNFi therapy. Typically, this reaction is not influenced by the underlying condition or the specific type of TNFi agents used, and it is resolved once the treatment is stopped. Therefore, this reaction may be considered as an effect of TNFi rather than a newly developed immune-mediated condition [9].

PP is divided into two types: new-onset psoriasis, which occurs in people with no previous history of psoriasis following TNFi agents for treating another inflammatory disease, and worsening or exacerbation of preexisting psoriatic lesions, with or without morphological differences. The first type is the most common and the most reported in the literature [25].

Clinically, PP may present several types of psoriasis ranging from plaque psoriasis (15.8–50%) to psoriasiform dermatitis (19.9%), guttate psoriasis (7–15%), generalized pustular psoriasis (5.3–12%), and inverse psoriasis (1.7–4%). Additionally, this paradoxical reaction can affect not only the skin but also the nails and scalp [26]. Whereas palmoplantar pustulosis (PPP) was described (33.3–45%), it may be considered a distinct entity [26], and it is necessary to make a distinction between classic psoriasis, PPP, and PP as seen in Table 1. 

According to the consensus statement from the European Rare and Severe Psoriasis Expert Network (ERASPEN), pustular psoriasis and psoriasis vulgaris are considered distinct phenotypes. Although they are clinically, immunologically, and genetically different entities, they can coexist: PPP may occur with or without psoriasis [27]. 

Palmoplantar pustulosis (PPP) is defined as a chronic, recurrent, inflammatory skin disease characterized by an eruption of sterile pustules with erythematous keratotic lesions that develop on the palms and soles and which usually is resistant to treatment [28]. The pathogenesis mechanism of PPP is not fully understood. Several studies have reported that the acrosyringium is the primary site where the inflammatory process in PPP lesions starts. The genetic susceptibility, variants in interleukin 36 receptor antagonist (*IL36RN*), adaptor related protein complex 1 subunit sigma 3 (*AP1S3)*, and caspase recruitment domain family member 14 *CARD14* genes), in additionally to environmental factors (smoking, metal allergies, female sex, autoimmune thyroid disease, stress, and infections) and dysregulation of multiple immunological pathways; in particular, the IL(interleukin)-17 and IL-36 pathways (with overexpression of IL-8) play an important role in the development of PPP [29].

The link between TNFi and PP was first reported in 2003 in a study involving 107 spondyloarthropathy patients on a TNFi agent (infliximab). Three patients with no personal or family history of psoriasis developed palmoplantar pustulosis [30].

The incidence of psoriasiform eruptions induced by TNFi therapy has been estimated to be between 3.5% and 10.7% [10], while prevalence rates have ranged from 0.6% to 5.3% [24,31] with a noted prevalence of 6% in IBD patients following TNFi therapy [32]. A higher prevalence was observed in patients with chronic inflammatory arthritic diseases [31].

**Table 1:** The differences between palmoplantar pustulosis, classic psoriasis, and paradoxical psoriasis.

Mechanism	Palmoplantar Pustulosis (PPP)	Classic Psoriasis	Paradoxical Psoriasis (PP)
**Genetic Factors**	Variants in *IL36RN*, ASP1S3 (rs2241880G and rs2241879A), and *CARD14* genes [28,29].	Variants in *HLA-Cw6*, *TNF*, *IL23R*, *IL12B*, *ERAP1*, *CARD14*, *TNFAIP3*, and *NFKBIA* [9,32].	Certain SNPs in genes like *IL23R*, *FBXL19*, *CTLA4*, *SLC12A8*, *TAP1*, and others predispose individuals to paradoxical psoriasis [22].
**Immunological pathways**	1. Dysregulation of multiple immunological pathways: - the IL-17 pathway, leading to excessive inflammation and skin lesions; - IL-36 pathways that lead to increased production of inflammatory mediators, contributing to the development and exacerbation of skin lesions; - overexpression of IL-8 can lead to an accumulation of neutrophils, which are a hallmark of pustular lesions [28]. 2. The role of the microbiome [28,29].	A dysregulation of certain immunological pathways, particularly the TNF/IL-23/IL-17 axis [9]	1. Cytokine dysregulation: TNFi blocks TNFα, which inhibits the maturation of cDCs and pDC, leading to an increased production of IFNα. The overexpression of IFNα leads to the failure of activation of autoimmune T cells and memory T cells [9,13,33,34]. 2. JAK-STAT pathway: TNFi blocks TNFα, leading to an overexpression of IFNα, which activates JAK1 and TYK2 and leads to the phosphorylation and activation of STAT1 and STAT2. Also, the elevated levels of IL-23 activate JAK2/TYK2, which activates STAT3, leading to Th17 cell differentiation and subsequent inflammation. This activation may have a potential role in the pathogenesis of PP [35,36]. 3. IL-23/Th17 axis involvement [33,37]. 4. TNF inhibitors may cause aberrant lymphocyte movement and increase CXCR3 ligands, such as CXCL9, CXCL10, and CXCL11, supporting a Th1-skewed inflammatory response and contributing to psoriasis lesions [38]. 5. Role of T cells and dendritic cells in the development of in paradoxical adverse events [39]. 6. Microbiota involvement and dysbiosis [40].
**Cytokine involved**	Increase in TNF-α, IL-22, IL17, and IFN-γ and increased expression of IL 8 [29].	Increased levels of TNF-α, IL-17, IL-23, and IL-22 [9].	Increased IFN-α, IL-17, and IL-22 [9].
**Environmental Triggers**	Smoking, stress, infections female sex, and autoimmune thyroid disease [29].	Trauma (Koebner phenomenon), infections, stress, and medications [41].	Infections, stress, smoking, female sex, and reaction to TNF inhibitors [42,43,44].
**Immune Cell Involvement:**	IL-17 plays an important role in the inflammation in PPP. Neutrophils are attracted to the skin by chemokines like IL-8 and play a significant role in the formation of pustules [45].	Hyperproliferation of keratinocytes; involvement of Th1, Th17, and Th22 cells, dendritic cells, and neutrophils [46].	− The involvement of pDCs producing IFN1; − T cells produce cytokines such as TNFα and IL-17, sustaining inflammation [9].
**Clinic presentation**	Sterile pustules on palms and soles [28].	Erythematous plaques characterized by silvery-white scales commonly on elbows, knees, scalp, and lower back [9].	Presence of different psoriatic patterns including plaque-type, guttate, and pustular forms [26].

AP1S3—adaptor related protein complex 1 subunit sigma 3, CARD14—caspase recruitment domain family member 14, cDCs—Conventional dendritic cells, CTLA4—cytotoxic T-lymphocyte associated protein 4, CXCL10—Chemokine ligand 10, CXCL11—Chemokine ligand 11, CXCL9—Chemokine ligand 9, CXCR3—chemokine receptor, ERAP1—endoplasmic reticulum aminopeptidase 1, FBXL19—(F-Box And Leucine-Rich Repeat Protein 19), HLA-Cw6—human leucocyte antigen, IFNα—Interferon alpha, IL12B—interleukin 12B, IL17—Interleukin-17, IL-23—Interleukin-23, IL23R—Interleukin 23 Receptor, IL23R—Interleukin 23 Receptor, IL36—Interleukin-36, IL36RN—interleukin 36 receptor antagonist, IL8—Interleukin-8, JAK1—Janus Kinase 1, JAK2—Janus Kinase 2, JAK-STAT Pathway—Janus kinase-signal transducer and activator of transcription Pathway, NFKBIA—NFKB inhibitor alpha, pDC—plasmacytoid dendritic cells, PPP—Palmoplantar Pustulosis, PP—paradoxical psoriasis, SLC12A8—solute carrier family 12 member 8, STAT1—Signal Transducer and Activator of Transcription 1, STAT2—Signal Transducer and Activator of Transcription 2, STAT3—Signal Transducer and Activator of Transcription 3, TAP1—transporter 1, ATP binding cassette subfamily B member, Th 1—T helper type 1 cells, Th 22 cells—T helper 22, Th17 cells—T helper lymphocytes, TNFAIP3—TNF alpha induced protein 3, TNFi—tumor necrosis factor inhibitors, TNF—tumor necrosis factor, TYK2—Tyrosine Kinase 2.

Certain risk factors, such as female gender, smoking, younger age at the start of administration of TNFi therapy, or using adalimumab or certolizumab, are highly associated with the risk of occurrence of psoriatic lesions during TNFi therapy in IBD patients [42,43,44]. A recent study involving 97 patients identified tobacco use and psychological stress, as well as a family history of psoriasis, as potential contributors to the development of PP [21,47].

### 2.2. Mechanisms Involved in PP

PP is a form of psoriasis that presents similar clinical characteristics to classical psoriasis but differs in its histological patterns and immunological processes [33]. 

Classical psoriasis is a chronic, inflammatory skin disease characterized by typical psoriatic skin and nail lesions and may be associated with a family history of psoriasis as well as the coexistence of other immune-inflammatory conditions. The underlying mechanism involves genetic predisposition (HLA-Cw6 is strongly associated with early onset severe psoriasis) and a dysregulation of certain immunological pathways, particularly the TNF/IL-23/IL-17 axis that has been shown to play a crucial role in the development of psoriatic lesions [9].

Generally, PP is described as an isolated reaction that mainly occurs following the treatment with TNFi drugs. However, as more biological agents are introduced and used to treat various inflammatory disorders, PP may become more prevalent [48]. 

The pathogenesis of this cutaneous reaction remains unclear, and various theories have been proposed [49], as seen in Table 2. Some theories explain the symptoms as a hypersensitivity reaction to TNFi rather than a newly developed disease. Vorčáková et al. noted in their study that increased production of antibodies against biologics can induce generalized pustulosis, which may be considered a hypersensitivity reaction [17,50]. However, other papers disprove this theory based on the skin biopsy of patients with PP. Also, it has been emphasized that certain genetic factors, such as the *IL23* receptor gene, have a significant impact on the induction of psoriasis lesions in ankylosing spondylitis or IBD (Crohn’s disease and ulcerative colitis) patients. These genetic factors also influence the immune response to biological treatments and contribute to the emergence of hypersensitivity reactions in some patients [17].

One of the most common and most widely accepted theories is the dysregulation in pro- and anti-inflammatory cytokine balance under the treatment with TNFi [13,51,52].

Biological agents, including TNFi or other targeted therapies, are designed to regulate cytokine levels by blocking specific cytokines like TNFα [53]. This blockade disrupts the balance of the immune system, leading to increased production of other cytokines and ultimately causing the development of inflammatory conditions [40]. This disruption in cytokine balance can lead to unexpected immune responses, such as PAEs [10,40,54].

Recent studies indicate that PP shares immunological features similar to those observed in the early phase of classic psoriasis [55] with the involvement of innate immunity systems, such as neutrophils, plasmacytoid dendritic cells (pDCs), macrophages, monocytes, and mast cells [55,56].

**Figure 1:**
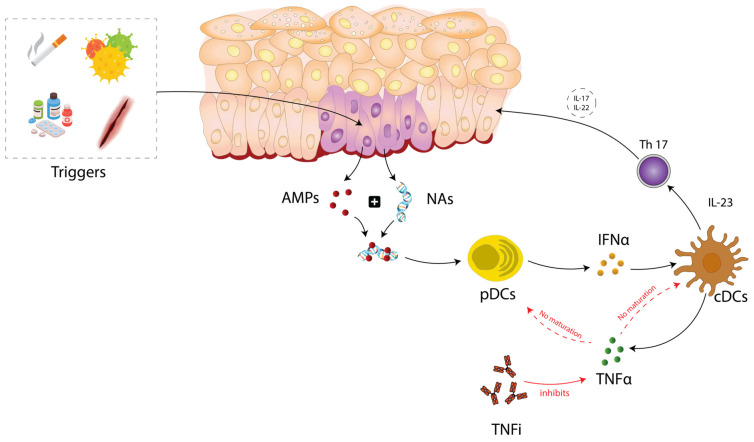
Schematic representation of the most common theories regarding the pathogenesis of paradoxical psoriasis (partial explanation) [9]. In classical psoriasis, a trigger factor (such as an injury to the skin, smoking, stress, or some drugs) leads to the production of antimicrobial peptides (AMPs) by keratinocytes. These AMPs form complexes with nucleic acids (NAs) which activate pDCs. These cells play an important role in the early phase of classic psoriasis pathogenesis, as they release IFNα. The early phase of classic psoriasis has similar immunological features to PP. Subsequently, IFNα activates cDCs, which produce TNFα and IL-23, which are crucial for the activation of autoimmune T cells. In PP, the use of TNFi disrupts this process. TNFi blocks TNFα, which is involved in the maturation and activation of dendritic cells and leads to unregulated IFNα production. In addition, without TNFα, cDCs cannot mature properly, which means they cannot elicit a T cell-mediated autoimmune response. This suggests that PP is independent of T cells, contrasting with the T cell-dependent mechanism seen in classical psoriasis [9]. The mechanism that connects IFNα overproduction to increased IL-17 and IL-22 production in the context of TNFi treatment is not fully established and may involve several immune pathways and cell types.

However, PP is predominantly mediated by the overexpression of pDCs-derived IFNα, which is in contrast to classic psoriasis that involves T cells [24,34]. In PP, TNFi blocks TNFα, which inhibits the maturation of conventional dendritic cells (cDCs) and pDC, leading to an increased production of IFNα as well as an increase in IL-23 and T-helper 17 cells. This activates intracellular antimicrobial procedures, which can affect immune responses [33] and potentially induce, in certain patients, the development of the disease [57]. As a result, the overexpression of IFNα leads to the failure of activation of autoimmune T cells and memory T cells, while classical psoriasis develops into a T-cell-mediated autoimmune process—Figure 1 [9,34].

Studies indicate that the IL-23/T-helper (TH)17 axis may be involved in developing PP in individuals with genetic predisposition [33,37]. IL-23 is a proinflammatory cytokine that promotes the activation of TH17 lymphocytes, which are involved in the pathogenesis of TNFi-induced psoriasis. The binding of IL-17 to its receptors stimulates the hyperproliferation and differentiation of keratinocytes, as well as the recruitment of immune cells to psoriatic lesions. TH17 cells produce IL-17, and their differentiation and activation are driven by IL-23. Consequently, blocking the IL-23/Th17 axis with an anti-IL-12/IL-23 antibody such as ustekinumab could effectively resolve skin lesions in IBD patients who develop PP [33,37].

A study from 2014 demonstrated that treatment with TNF inhibitors (TNFi) might lead to an increased production of TH1 and TH17 cells. This increase results in a higher number of IFN-γ-secreting TH1 cells and IL-17/IL-22-secreting TH17 cells in patients who develop psoriasis after TNFi therapy. These findings suggest a link between the use of TNFi medication and elevated levels of cytokines IL-17 and IL-22 [38,58]. This correlation is further supported also by Jiraskova Zakostelska et al., who showed that IBD patients with psoriasis lesions following TNFi therapy had higher levels of IL17A and IL23 compared to those without such lesions [40]. Cutaneous paradoxical reactions, particularly PP, are characterized by the infiltration of IL17A/IL22-expressing TH17 cells and IFNα-secreting TH1 lymphocytes [59], with the reaction’s severity linked to the density of TH17 cell infiltration in the skin [38].

However, all these studies underline the need for further research to fully understand the mechanisms through which anti-IL-12/IL-23 treatment operates in the context of cutaneous paradoxical reactions.

Another potential mechanism involved in paradoxical psoriasis is the JAK/STAT pathway. The activation of some JAK family members (JAK1, JAK2, and TYK2) is initiated by cytokines such as IFN-γ, IL-6, and those involved in the IL-23/IL-17 axis, which are known to drive the pathogenesis of psoriasis by promoting inflammation, immune cell activation, and abnormal keratinocyte growth. It suggests that blocking TNF-α may lead to uncontrolled upregulation of type 1 interferons, which activate JAK1/TYK2 and downstream STAT1/STAT2 pathways, which then drive the expression of pro-inflammatory genes and result in excessive inflammation. Similarly, TNF-α inhibition dysregulates IL-23, activating JAK2/TYK2 and STAT3, which promotes Th17 cell differentiation and activation, leading to the release of IL-17 and IL-22 and causing cutaneous inflammation. The activation of the JAK-STAT pathway by these cytokines contributes to the chronic inflammatory state and the development and exacerbation of psoriatic lesions [35,36].

It has also been suggested that TNFi may cause an aberrant movement of lymphocytes and increase the production of CXCR3 ligands, which are involved in the development of psoriatic lesions [38]. The overexpression of IFNα stimulates the expression of CXCR3 ligands, such as CXCL9, CXCL10, and CXCL11. These ligands can then support a Th1-skewed inflammatory immune response, which induces the recruitment of CXCR3+ cytotoxic T lymphocytes in skin lesions, further contributing to the development of psoriasis lesions [60,61]. A study conducted in 2009 involving 13 patients with psoriasis induced by TNFi drugs found that there was a higher expression of CXCL9 in the epidermis of psoriasiform lesions than in healthy control skin and psoriasis patients. Additionally, CXCR3 and TIA1 were found to be highly expressed in psoriasiform eruption infiltrates, which suggests that the increased expression of CXCR3 and its ligands may have a role in the occurrence of psoriasiform eruptions in patients treated with TNFi [60]. However, few studies examined the association between paradoxical psoriasis and CXCR3 ligands [58].

Besides cytokine dysregulation, alterations in the function and signaling of immune cells such as dendritic cells (DC) or T cells are also implicated in the development of PAEs. Chronic immune-mediated inflammatory diseases involve various immunological pathways, and the use of biological agents as treatment can alter the cytokine milieu, leading to the development of new pathways that facilitate a favorable immunological context for the occurrence of PAEs [9,38].

Therefore, both T cells and DC are involved in the occurrence of PAEs: T cells play an important role in the pathogenesis of psoriasis by producing cytokines, for example, TNFα and IL-17, which sustain inflammatory mechanisms for psoriasis lesions, while dendritic cells are key producers of IFNα.

T cells are a type of lymphocyte that plays a crucial role in adaptive immune responses, and any dysregulation of T cells is strongly associated with inflammatory and autoimmune diseases, such as psoriasis [39].

TNFα has a significant impact on T cell function. TNFα can enhance T cell activation, proliferation, and cytokine production, which can promote immune responses.

TNFi agents also play an important role in modulating T cell function by inhibiting TNFα. TNFi has various effects on T cells, including the suppression of T cell activation pathways and the downregulation of pro-inflammatory cytokines production. It is crucial to recognize that altering T cells’ response through TNF inhibition can also present potential disadvantages or risks [62,63], such as the development of PAEs.

DCs are a type of immune cell that acts as professional antigen-presenting cells, which means they capture, process, and present antigens to T cells, which ultimately leads to the activation and shaping of immune responses. TNFα modulates DC function by influencing their maturation, survival, migration, antigen presentation, and cytokine production. The balance between immature and mature DC is important for maintaining a healthy immune system. Immature DC induces T-cell tolerance, while mature DC activates T-cells, promoting immune responses. However, the excessive enhancement of DC survival can disrupt immune tolerance, which may lead to the occurrence of autoimmune diseases [64].

By inhibiting TNFα, TNFi induces, in healthy tissue, alterations of the balance of cytokines (TNFα/IFNα) and activates DC, which can consequently trigger a paradoxical reaction [65].

Another mechanism that could be involved in the development of PP is dysbiosis. Changes in the gut or skin microbiota might influence systemic inflammation, contribute to the pathogenesis and progression of IBD or psoriasis, and trigger or exacerbate psoriasis, including paradoxical forms [40]. Skin–gut interaction is facilitated by several mechanisms, and the microbiota plays a central role in this process. The correlation between psoriasis and Crohn’s disease (CD) is widely recognized. Any modifications in the composition of the microbiota can potentially result in an immune system response alteration, ultimately leading to the development of inflammatory diseases [66,67]. Biologics, particularly TNFi, are widely accepted for treating IBD and psoriasis patients. The current literature indicates that biological treatment can influence the microbiota’s composition, and certain patients may not respond efficiently to this treatment because of potential variations in microbiota. Changing in the composition of microbiota leads to changes in microbiota interactions, which subsequently affects the immune system and alters the body’s homeostasis. The close relationship between psoriasis and IBD has also been highlighted by the discovery that one of them can manifest as a paradoxical adverse event under the treatment of TNFi for the other one [40]. Studies reported that CD patients have an augmented risk of developing psoriasis (about five times more) compared with the general population. Also, patients with psoriasis have a greater risk of developing CD [66]. To improve therapy management and reduce side effects, it is essential to understand the role of the microbiota in paradoxical responses to targeted therapies [40]. However, the exact molecular mechanisms of this host–microbe interaction are still unknown.

The different pathophysiological mechanisms of PP discussed in this article emphasize the necessity for further research, as the specific immune pathways contributing to this condition are still not fully understood.

## 3. Genetic Factors and Paradoxical Psoriasis

Recent studies have highlighted that genetic predisposition, apart from predicting the efficacy of the therapeutic responses, has a central role in the pathogenesis of various autoimmune disorders [68] or the development of various PAEs [22,69], especially PP. Many “susceptibility” genes are associated with various diseases, regulating common inflammation pathways [17].

In the current body of literature, some genes have been investigated for their potential association with the increased risk of various chronic immune-inflammatory diseases or PP [3,22,70]. For example, certain genetic variants of the *IL23R* may contribute to the increased incidence of psoriasis in ankylosing spondylitis, ulcerative colitis, and Crohn’s disease patients. However, in the literature, limited data exists about the involvement of gene polymorphisms in the development of PP [17]. SNP is the most frequent variation in the DNA sequence of a single base at a specific location in the genome that differs between individuals [71]. A variation at a single base pair location in the genome is classified as an SNP if at least two different versions (alleles) of that location are found in over 1% of a large, unrelated population [72].

The “rs” number is a unique identifier assigned to a specific SNP in the genome. It helps researchers and scientists to study and reference specific genetic variations across different populations and studies. Each “rs” number corresponds to a specific location on a chromosome where the SNP is located.

The genetic variations not only influence susceptibility to certain diseases but may also act as prognostic markers for therapeutic responses and the development of side effects, including the appearance of psoriasiform lesions following the TNF blockade.

Few studies have investigated the potential association between some SNPs (such as *rs10789229*, *rs1799964*, and *rs11209026*) as potential markers and the increased risk of PP [3,22,70]. The correlation between these SNPs and PP suggests that genetic factors may play an important role in the occurrence of these types of reactions.

A comprehensive literature research was conducted using EMBASE, EBSCO, and PubMed databases to report the genes implicated in the development of PP in pediatric and adult patients after the administration of treatments with TNFi. Overall, only five articles were identified, including one pediatric study and four adult studies. All these studies are made only on IBD, psoriatic, or HS patients. There were no publications on genes associated with PP in rheumatological patients treated with TNFi. The genes *IL23R*, *FBXL19*, *CTLA4*, *SLC12A8*, and *TAP1* have been studied for their role in the development of PP [9,55]. All these studies are summarized in Table 3.

A number of SNPs of different genes were found to be associated with TNFi-related PP, including certain SNPs of the *IL23R*, *FBXL19*, *CTLA4*, *TAP1*, and *SLC12A8* genes. Notably, the studies focusing on the genetic findings in TNFi-related PP included groups of patients who varied with respect to age (pediatric or adult populations), diagnosis (IBD, IBD, and psoriasis), and treatment (infliximab, adalimumab, and etanercept), as well as the investigated genes [3,22,38,55,69].

In 2013, Sherlock et al. investigated the association between the *IL23R* gene and the occurrence of PP in IBD patients treated with infliximab therapy [69]. The authors specifically looked at some SNPs, including *rs10489628*, *rs1343151*, *rs10789229*, *rs2201841*, and *rs11209026*, and tried to find whether there was any correlation between these genetic variations and the development of infliximab-induced psoriasis. They made a study on a smaller number of pediatric IBD patients, suggesting that *IL23R* genes (*rs10489628*, *rs10789229*, and *rs1343151*) were more prevalent in patients with psoriasis after infliximab therapy than CD patients treated also by infliximab but who did not develop psoriasis [69]. The study also found that *rs10789229* has the highest risk of paradoxical psoriasis compared to rs2201841 and rs11209026, which did not show any association.

Another study conducted by Tillack et al. genotyped 17 SNPs associated with the *IL23R* and *IL12B genes* (*rs1004819*, *rs7517847*, *rs10489629*, *rs2201841*, *rs11465804*, *rs11209026*, *rs1343151*, *rs10889677*, *rs11209032*, *rs1495965*, *rs12131065*, *rs7530511*, *rs3212227*, *rs6887695*, *rs2082412*, *rs10045431*, and *rs2066808*) in IBD patients who developed TNFi-induced skin lesions [38]. The study found that the *rs11209026* (especially p.Arg381Gln) and *rs7530511* (p.Leu310Pro) variants may have some disease-modifying effects in patients with severe lesions who require ustekinumab therapy. Additionally, the study demonstrated that individuals who carry the wildtype of the *rs11209026* SNP are associated with an increased Th17 cytokine production. Conversely, individuals carrying the minor allele exhibit a reduced secretion of Th17 cytokines, including IL-22 [38].

According to this study, all patients with severe psoriasiform skin lesions and/or anti-TNF-induced alopecia who require ustekinumab therapy were found to have G/G wildtype carriers for the rare coding gene *IL23R* variant *rs11209026* (p.Arg381Gln). Furthermore, there was a higher prevalence of individuals carrying the C/T heterozygote form of the *rs7530511 (IL23R* gene) among patients with severe skin lesions who require ustekinumab treatment than IBD patients treated with TNFi who do not have skin lesions. The difference was statistically significant (42.9% vs. 21.6%; *p* = 0.05). This indicates that these genetic variations could influence the disease course in patients suffering from severe psoriasis induced by TNFi [47].

An important study conducted by Cabaleiro et al. involved 161 patients with plaque-type psoriasis treated with TNFi. Of these, a subset of 25 patients (11 men and 14 women) experienced PP and were genotyped for 173 SNPs [22]. The goal of the genotyping analysis was to study the role of genetic polymorphisms in the development of PP among patients with psoriasis. Five specific SNPs were identified by the researchers as being associated with the occurrence of paradoxical reactions: *rs11209026* in *IL23R*, *rs10782001* in *FBXL19*, *rs3087243* in *CTLA4*, *rs651630* in *SLC12A8*, and *rs1800453* in *TAP1*.

According to this study, the researchers found that the protective allele A in *rs11209026* affects IL-23 signal transduction in a negative way, leading to a decrease in IL-17A. They observed that the A allele was more frequent in patients with paradoxical reactions after TNFi drugs. This finding suggests that the *rs11209026* of the gene *IL23R* may contribute to the occurrence of PP following TNFi treatment. *IL23R* was the only gene that has been previously associated with psoriasis and other autoimmune diseases. The presence of the A allele in this SNP may affect the balance of cytokines involved in the pathogenesis of psoriasis and contribute to the development of paradoxical reactions. However, this study provides valuable insights into the potential role of genetic factors in modulating the risk of paradoxical reactions in patients receiving anti-TNFα therapy [22].

In 2019, Fania et al., in their study, investigated the genetic and immunological profiles of three patients with hidradenitis suppurativa (HS) who developed PP following TNFi therapy (Adalimumab). Similar to previous research, they found that PP has immunological characteristics similar to the early phases of classic psoriasis development, including the significant presence of innate immunity cells such as neutrophils, plasmacytoid dendritic cells, mast cells, macrophages, and monocytes. Additionally, the study analyzed some SNPs and genes, including *ERAP1*, *NFKBIZ*, *TNFAIP*, and *HLA-C* genes, predisposing to psoriasis in HS patients who develop PP under TNFi. All patients with HS had variations in the *ERAP1* gene (*rs30187*/*rs30186*/*rs26653*) and the *HLA-C* region (*rs114395371*/*rs9264942*/*rs10484554*/*rs2524095*/*rs28383849*/*rs9264944*/*rs2853922*/*rs147538049*/*rs9264946*). The *HLA-Cw6* allele, strongly associated with psoriasis, was not present, but other nearby variants were. All three patients with HS and PP have variations in the *NFKBIZ* and *TNFAIP3* genes which may contribute to NF-κB hyperactivation in patients who develop PP. Additionally, three SNPs in the *IL23R* gene were analyzed in patients with PP. One of the SNPs in the *IL23R* gene, *rs11209026*, was earlier linked with PP in patients treated with TNFi. However, in this study, this SNP was not detected in any of the three patients with HS. Two SNPs of the *IL23R* gene, *rs72676067* and *rs1004819*, were identified in patients 2 and 1. Furthermore, patient 3 presented several SNPs associated with psoriasis. Additionally, he had some SNPs (*rs7637230*, *rs4819554*, *rs3132554*, *rs10542126*, *rs3130983*, and *rs280519*) in common with patient 2, as well as one SNP *(rs280519*) with patient 1. Overall, the study highlights the need for further research to fully understand the genetic and molecular mechanisms underlying this condition [55].

In 2020, Bucalo et al., in their study, investigated the association between specific genetic variations and disease status in 161 psoriasis and IBD patients treated with TNFi drugs. They genotyped the patients for six SNPs that were selected based on their potential susceptibility to classical psoriasis and PP, as well as based on the response to TNFi agents. The six SNPs that were analyzed in the study included *rs10484554* (*HLA-Cw0602* gene), *rs11209026* and *rs10789229* (*IL23R* gene), *rs1799964* and *rs1800629* (*TNFα* gene), and *rs1990760* (*IFIH1*). This study investigated the variations of genotype frequencies of particular SNPs in IBD and psoriasis patients with and without PP. The results showed a statistically significant variation in the genotype distribution of the TNFα gene, specifically for SNP *rs1799964*, among patients with IBD and PP. These patients had a higher probability of carrying the rare C allele of this SNP compared to those without PP. This suggested that the rs1799964 variant of the TNFα gene may be implicated in the onset of PP in individuals with IBD [3].

Also, no statistically significant associations were identified for rs10484554 of the *HLA-Cw06* gene and *rs10789229* of *IL23R* when comparing patients with psoriasis, both with and without PP. This indicates that these specific SNPs may not be directly correlated with the occurrence of PP in psoriatic patients.

All these studies highlight not only the complex and the importance of the relationship between genetic variations, especially in some genes such as *TNFα*, *IL23R*, *FBXL19*, *CTLA4*, *SLC12A8*, and *TAP1*, and the development of PP in patients treated with TNFi but also the potential of using these markers to predict susceptibility to PP in patients experiencing TNFi therapy.

However, there are some clinical limitations. Diagnosing and treating PP represents a challenge to clinicians, considering the heterogeneous groups of patients and the complex interactions of various medications. PP may appear several months after starting a medication, and a full medical history is mandatory for a correct diagnosis. A close collaboration between rheumatologists and dermatologists is important for understanding, diagnosing, and treating this condition. A correct clinical and histopathological examination is required to distinguish PP from preexisting psoriasis or another rare manifestation of an autoimmune disease.

Several studies included a limited number of cases with varied demographic characteristics and various immune-mediated inflammatory diseases. This diversity complicates accurate patient classification and assessment of the true impact of TNF inhibitors on PP development.

In conclusion, despite its clinical significance, this condition remains under-researched, poorly understood, thus requiring further investigation.

## 4. Conclusions

TNFi have become a benchmark for the treatment of various immune-mediated inflammatory diseases, including IBD, psoriasis, and rheumatologic conditions, having a great impact on improving symptoms and the patients’ quality of life. Although the TNFi treatment is well tolerated, 2–5% of patients develop paradoxical adverse effects following this treatment. The mechanism of this type of reaction remains unclear, and several theories have been proposed to explain this immune phenomenon. Some authors suggested that genetic factors also play an important role in the development of PAEs. Certain authors noted that there is a complex relationship between genetic polymorphism and PAEs, especially PP. It is important to identify which SNPs are associated with the occurrence of PP. Knowledge of these genetic markers is important, not only for understanding the underpinnings of PP but also for the use of personalized treatment strategies, possibly allowing clinicians to identify patients at higher risk of developing PAEs in future years. Integrating genetic insights into clinical practice may represent a significant step towards personalized medicine, as it could allow healthcare providers to refine their therapeutic strategies, optimizing treatment efficacy while concurrently minimizing the potential for these adverse reactions.

## Figures and Tables

**Table 2:** Potential factors involved in the pathogenesis of paradoxical psoriasis.

Mechanism	Description	References
Cytokine dysregulation	Dysregulation in cytokine balance is one of the most common and most accepted theories. In PP, TNFi blocks TNFα, which inhibits the maturation of conventional dendritic cells (cDCs) and pDC, leading to an increased production of IFNα. As a result, the overexpression of IFNα leads to the failure of activation of autoimmune T cells and memory T cells.	Mylonas A et al., 2018 [9] Toussirot É et al., 2016 [13] Lu J et al., 2023 [33] Conrad C et al., 2018 [34]
JAK-STAT pathway	TNFi blocks TNFα, leading to an overexpression of IFNα, which binds to its specific receptors on cells, activates JAK1 and TYK2, and leads to the phosphorylation and activation of STAT1 and STAT2. These induce the expression of proinflammatory genes, resulting in amplified inflammation and potentially explaining the development of psoriasis. Also, the elevated levels of IL-23 activate JAK2/TYK2, which activates STAT3, leading to Th17 cell differentiation and subsequent inflammation. This activation may have a potential role in the pathogenesis of PP.	Woodbury MJ et al., 2024 [35] Zhang Y et al., 2024 [36]
IL-23/Th17 axis involvement	IL-23 promotes the activation of Th17 cells, which produce IL-17 and IL-22. Blocking the IL-23/Th17 axis with anti-IL-12/IL-23 antibodies like ustekinumab can resolve skin lesions in IBD patients with paradoxical psoriasis.	Lu J et al., 2023 [33] Wasilewska A et al., 2021 [37]
CXCR3 ligands and T cell migration	TNF inhibitors may cause aberrant lymphocyte movement and increase CXCR3 ligands, such as CXCL9, CXCL10, and CXCL11, supporting a Th1-skewed inflammatory response and contributing to psoriasis lesions.	Tillack C et al., 2013 [38]
Role of T cells and dendritic cells	T cells produce cytokines such as TNFα and IL-17, sustaining inflammation, while dendritic cells produce IFNα. Both play crucial roles in paradoxical adverse events.	Kumar BV et al., 2018 [39]
Genetic factors	Certain SNPs in genes like *IL23R*, *FBXL19*, *CTLA4*, *SLC12A8*, *TAP1*, and others predispose individuals to paradoxical psoriasis.	Cabaleiro T et al., 2015 [22]
Microbiota involvement and dysbiosis	Changes in the gut or skin microbiota might influence systemic inflammation, contribute to the pathogenesis and progression of IBD or psoriasis, and could trigger or exacerbate psoriasis, including paradoxical forms. Certain modifications in the composition of the microbiota can potentially result in an immune system response alteration, ultimately leading to the development of an inflammatory response.	Zákostelská Z J et al., 2023 [40]

cDCs—conventional dendritic cells, CTLA4—cytotoxic T-lymphocyte-associated protein 4, CXCL—chemokine ligand, CXCL9—chemokine ligand 9, CXCR3—chemokine receptor, FBXL19—F-Box And Leucine-Rich Repeat Protein 19, IBD—inflammatory bowel diseases, IFNα—Interferon alpha, IL—interleukin, IL23R—Interleukin 23 Receptor, JAK-STAT pathway—Janus kinase-signal transducer and activator of transcription pathway, pDC—plasmacytoid dendritic cells, PP—paradoxical psoriasis, SLC12A8—solute carrier family 12 member 8, SNPs—Single Nucleotide Polymorphisms, TAP1—transporter 1, ATP binding cassette subfamily B member, Th—T helper lymphocytes, TNFi—tumor necrosis factor inhibitors, TNFα—tumor necrosis factor alpha, TYK2—Tyrosine Kinase 2.

**Table 3:** Genetic factors associated with paradoxical psoriasis.

Article	Patients	Number of Patients	Treatment	Gene	SNPs	Association with PP
Sherlock M.E et al., 2013 **[69]**	Pediatric patients with IBD and psoriasis	Total of 234 IBD patients (147 with CD, 87 with UC). Total of 35 patients used Infliximab treatment. Total of 10 developed PP under Infliximab.	IFX	*IL23R*	rs2201841 rs10489628 rs10789229 rs11209026 rs1343151	Not associated Associated Associated Not associated Associated
Tillack C et al., 2014 **[38]** *	IBD patients	Total of 434 patients with IBD. Total of 21 develop PP (16 under IFX, 5 under ADA).	IFX or ADA	*IL23R* *IL12B* *IL23A*	rs1004819 rs7517847 rs10489629 rs2201841 rs11465804 rs11209026 (*p.Arg381Gln*) rs1343151 rs10889677 rs11209032 rs1495965 rs12131065 rs7530511 (*p.Leu310Pro*) rs3212227 rs6887695 rs2082412 rs10045431 rs2066808	Not associated Not associated No association No association No association Disease-modifying effects No association No association No association No association No association Disease-modifying effects No association No association No association No association No association
Cabaleiro T et al., 2015 **[22]**	Psoriasis patients	Total of 161 patients with moderate to severe plaque psoriasis. Total of 25 develop PP (18 under ETA, 5 under ADA, 2 under IFX).	TNFi (ETA, ADA, IFX)	*IL23R*	rs11209026	Associated
				*FBXL19*	rs10782001	Associated
				*CTLA4*	rs3087243	Associated
				*SLC12A8*	rs651630	Associated
				*TAP1*	rs1800453	Associated
Fania L et al., 2019 **[55]** **	HS	patients with HS	ADA	*ERAP1*	rs30187	Possibly associated
					rs30186	Possibly associated
					rs26653	Possibly associated
					rs11743410	Possibly associated
				*HLA-C*	rs114395371	Possibly associated
					rs2524095	Possibly associated
					rs2853922	Possibly associated
					rs386698994	Possibly associated
					rs28383849	Possibly associated
					rs10484554	Possibly associated
					rs147538049	Possibly associated
					rs9264944	Possibly associated
					rs9264946	Possibly associated
				*HLA-Cw6 2v*	rs79709508	Possibly associated
				*HLA-Cw6*	rs17192540	No association
				*IL23R*	rs72676067	Possibly associated
					rs1004819	Possibly associated
					rs41313262	Possibly associated
					rs11209026	No association
				*NFKBIZ*	rs3217713	Associated
					rs7637230	Possibly associated
				*IL12B*	rs2546890	Possibly associated
				*TNF α*	rs1800610	Possibly associated
				*IL17F*	rs2397084	Possibly associated
				*TRAF3IP2*	rs71562288	Possibly associated
					rs33980500	Possibly associated
				*TNFAIP3*	rs610604	Possibly associated
				*TYK2*	rs12720356	Possibly associated
					rs280519	Possibly associated
				*IL17RA*	rs4819554	Possibly associated
				*CDSN*	rs3132554	Possibly associated
					rs1042127	Possibly associated
					rs1042126	Possibly associated
					rs1062470	Possibly associated
					rs707913	Possibly associated
					rs3130983	Possibly associated
				*CCHCR1*	rs1576	Possibly associated
					rs130079	Possibly associated
					rs746647	Possibly associated
					rs130075	Possibly associated
Bucalo A et al., 2020 **[3]**	IBD and psoriasis patients	Total of 53 IBD patients. Total of 16 develop PP (11 under ADA, 5 under IFX). Total of 108 psoriatic patients. Total of 23 develop PP under ADA.	TNFi (ADA, IFX, ETA)	*HLA-Cw06*	rs10484554	Not associated
				*IL23R*	rs11209026	Associated
					rs10789229	Not associated
				*TNFα*	rs1799964	Associated
					rs1800629	No association
				*IFIH1*	rs1990760	No association

* palmoplantar pustulosis was regarded as PP and analyzed together with other types of PP. ** all patients developed both erythematosquamous psoriasis lesions and pustulosis.
